# The Impact of Tourniquet Usage on TKA Outcome: A Single-Center Prospective Trial

**DOI:** 10.3390/medicina59050870

**Published:** 2023-04-30

**Authors:** Serban Dragosloveanu, Christiana Dragosloveanu, Mihnea Petre, Mihai E. Gherghe, Dragos C. Cotor

**Affiliations:** 1 Department of Orthopaedics, “Foisor” Orthopaedics Hospital, 030167 Bucharest, Romania; 2 “Carol Davila” Faculty of Medicine, University of Medicine and Pharmacy, 050474 Bucharest, Romania; 3 Clinical Hospital for Ophthalmological Emergencies, 030167 Bucharest, Romania

**Keywords:** tourniquet, TKA, osteoarthritis

## Abstract

*Background and Objectives*: Total knee arthroplasties (TKAs) are the most effective surgical treatment for end-stage knee osteoarthritis. The tourniquet is used to reduce intraoperative blood loss, improving surgical field visualization. There is much controversy regarding the effectiveness and safety of using a tourniquet during total knee arthroplasties. The purpose of this prospective study is to determine the effect of tourniquet usage during TKAs on early functional outcomes and pain in our center. *Materials and Methods*: We conducted a randomized controlled trial of patients following a primary total knee replacement between October 2020 and August 2021. We recorded presurgical data, which included age, sex and knee range of motion. Intraoperatively, we measured the amount of blood aspiration and the surgical room time. After the surgery, we measured the amount of blood aspirated through the drains and the hemoglobin. We measured flexion, extension, Visual Analogue Scale (VAS) scores, and Western Ontario and McMaster Universities Arthritis Index (WOMAC score) scores for the functional evaluation. *Results*: We included 96 patients in the T group and 94 in the NT group, respectively, who remained until the last follow-up. Regarding blood loss, the NT group demonstrated significantly lower levels: 245 ± 97.8 mL intraoperative and 324.8 ± 151.65 mL postoperative, compared to the T group, where we recorded 276 ± 109.2 mL during the surgical procedures and 353.44 ± 101.55 mL after the surgery, (*p* < 0.05). We also recorded significantly shorter operative room time for the NT group, (*p* < 0.05). During the follow-up, we noticed postoperative improvements but without significant differences between the groups. *Conclusions*: We found a significant decrease in bleeding after no tourniquet usage during total knee replacements and shorter operative times. On the other hand, the knee function demonstrated no significant differences between the groups. Further studies may be required in order to assess complications.

## 1. Introduction

Total knee arthroplasties (TKAs) are the most effective surgical treatment for end-stage knee osteoarthritis patients and one of the most widespread orthopaedic surgical procedures; over six million cases per year are projected in the following decades [1,2]. However, this invasive procedure is associated with significant blood loss, which in some cases would require a blood transfusion. For this reason, there is a growing need for improvements in operative room efficiency and the quality of surgical treatment [3]. An essential step in total knee arthroplasties, and is now accepted as a standard method during TKAs, is the use of a tourniquet [4,5,6,7]. The tourniquet is used to reduce intraoperative blood loss, improving the visualization of the surgical field, and it is believed that it may provide better cementation with the bone interface [8,9,10,11,12]. The tourniquet may also provide a more straightforward surgical procedure due to a bloodless field. It is also believed that tourniquet usage also allows better integration of the cement into the bone [9,13]. Better cementing reduces the risk of prosthetic loosening. On the other hand, the clinical evidence regarding this subject still needs to be clarified, as many reports in the literature demonstrate mixed results [14,15,16,17,18].

There is much controversy regarding the effectiveness and safety of using a tourniquet during a total knee arthroplasty. In addition, this surgical maneuver also raises questions regarding postoperative pain, the risk of thrombosis and postoperative recovery. Patients who have a tourniquet applied during surgery very frequently complain of thigh pain caused by direct pressure on local soft tissues and nerves after the tourniquet usage. When a tourniquet is applied, the mechanism of the quadriceps is altered, and intraoperative patellar tracking is modified. It seems that the usage of a tourniquet for over 60 min upregulates the proteolytic activity within muscles, which may explain the negative effect on functional outcomes after a total knee replacement. Limb swelling and increased soft tissue tension caused by reactive hyperperfusion after tourniquet deflation may also contribute to local pain [19,20]. Moreover, a recent paper claimed that no significant differences had been recorded regarding pain scores and level of opioid consumption, and it also did not find an impact on the outcomes [14].

One of the most severe postoperative complications in orthopedic surgery is pulmonary thromboembolism, which is also a significant cause of death. It has been found that 1% of orthopaedic cases report venous thromboembolism, among which 7.1% are fatal [21,22,23]. The deflation of a tourniquet changes the hemodynamic status, which may allow a substantial amount of potential emboli to travel from the lower limb. It has also been reported that postoperative cognitive deficits may be caused by systemic emboli after a total knee replacement [24]. Other tourniquet-related complications reported in the literature include vascular lesions, a decreased range of motion, rhabdomyolysis, vascular paralysis, swelling and subcutaneous fat necrosis of the thigh caused by local hypoxia [9,11,25,26,27]. Therefore, the benefits of tourniquet usage should be taken into account, but not without considering its risks. Recently, among orthopedic surgeons, total knee arthroplasty without a tourniquet has been increasingly used and reported in several papers. Thus, this is not a novel subject, even though it is controversial [15,16,17].

However, since the evidence regarding this topic from our country is lacking and needs to be improved, the main objective of this prospective study is to evaluate if tourniquet usage during TKAs has an effect on early and midterm functional outcomes and pain in our center. We also wanted to evaluate if this technique has an impact on operative time and blood loss. The hypothesis of this study was that total knee arthroplasty without a tourniquet positively influences postoperative functional outcomes, total blood loss and patient satisfaction.

## 2. Materials and Methods

### 2.1. Design

We conducted a randomized controlled trial of patients following a primary total knee replacement between October 2020 and August 2021 in our center. We excluded from our study patients with revisions and cases requiring a greater constraint than a posterior stabilized TKA (constrained or hinged knee). We also excluded patients with coronary diseases, neurological diseases, infectious conditions, coagulation disorders or a history of deep vein thrombosis or pulmonary embolism. Inclusion criteria consisted of patients aged between 50–80 years and with BMI < 45 kg/m^2^. Patients included in our assessment were split into two groups, one which underwent a total knee replacement using a tourniquet (T) and one without a tourniquet (NT). The randomization was made by an independent doctor using sealed envelopes. The envelopes were drawn before the procedure and specified the surgical technique. This paper was approved by the “Foisor” Orthopaedics Clinical Hospital Ethics Committee of Bucharest, Romania, approval number 2020/2316 (date of approval 10 July 2020). This paper was designed and developed in agreement with the Helsinki Declaration. All patients provided written informed consent before the procedure.

### 2.2. Surgical Technique

All TKA procedures were performed by a single, highly experienced orthopedic surgeon in our institution, who specializes in total knee arthroplasty, according to a standard protocol. The implant of choice was the Zimmer Biomet NexGen implant system. Preoperatively, 1 g of tranexamic acid was administered in all cases, with an additional 1 g administered after the procedure. All cases received spinal anesthetic with a femoral nerve block. For the T group cases, a tourniquet was installed after three minutes of elevating the lower limb, which was inflated up to 350 mm Hg prior to the incision and deflated after the implantation, in order to perform hemostasis. 

We used a parapatellar approach, an intramedullary femoral guide with posterior referencing, an extramedullary tibial guide and a measured resection technique. For all cases, we routinely resurfaced the patella. All cases received a deep drain, and the wound closure was performed in a layered fashion. The drain was clamped for 3 h and then released and removed 24 h after surgery. Patients began physical therapy the following day and received chemoprophylaxis for deep vein thrombosis. Physical therapy was continued either at home or in a specialized clinic.

We recorded preoperative data, which included age, sex, knee range of motion and Western Ontario and McMaster Universities Arthritis Index scores (WOMAC Score). Intraoperatively we measured the amount of blood aspiration by taking into account the amount of physiological serum used for lavage and the surgical room time. After the surgical procedure, we measured the amount of blood aspirated through the drains and the hemoglobin. For the functional evaluation, we measured flexion, extension, Visual Analogue Scale (VAS) scores and WOMAC scores after 6 weeks and 6 months. All complications were recorded throughout the whole study.

### 2.3. Outcome Measures

The Western Ontario and McMaster Universities Osteoarthritis Index (WOMAC) was the primary outcome measure of this paper; this is a questionnaire consisting of 3 subsections (pain, stiffness and function) and comprises 24 items [28].

The Visual Analogue Scale is a measure of pain to determine the patient’s perspective of pain level. It may be dependent on the patient’s mental status, but it is very frequently used to evaluate and correlate knee function [29,30].

Both measures were translated and provided for patients to answer in the hospital and during follow-up visits.

### 2.4. Statistical Analysis

Continuous variables were normally distributed and were presented as means and standard deviations. A statistical analysis was performed by using an independent t-test. Categorical data were analyzed using the Chi-square test. All of the statistical analysis was performed by an independent statistician using SPSS version 26.0 (IBM Corp., Armonk, NY, USA), and we established a level of *p* < 0.05 as statistically significant. Even though bleeding was also a point of assessment, our main objective was to assess knee function during our study. Thus, we performed a power analysis for outcome measures of VAS and WOMAC scores. This was performed based on other published data about functional recovery and outcome measures available in the literature [10,31,32]. Using an alpha error of 0.05, a statistical power of >0.8 and a medium effect size of 0.5, we determined that a minimum of 180 cases were required. By considering the dropout risk, we enrolled 200 patients, who were split equally between the two groups. 

## 3. Results

We included 96 patients in the T group and 94 in the NT group, respectively, who remained part of this study until the last follow-up. All 10 cases that dropped out have been excluded. Regarding patient age and gender, we did not identify any significant differences. (Table 1) Preoperative hemoglobin and knee range of motion also showed no statistically significant differences.

Regarding blood loss, the NT group demonstrated significantly lower levels: 245 ± 97.8 mL intraoperative and 324.8 ± 151.65 mL postoperative, compared to the T group, where we recorded 276 ± 109.2 mL during the surgical procedures and 353.44 ± 101.55 mL after the surgery, (*p* < 0.05). Regarding postoperative hemoglobin, the Tourniquet group demonstrated significantly lower values (11.23 ± 1.5) compared to the No Tourniquet group (11.5 ± 1.3), (*p* < 0.05). We also recorded significant differences in operative room time between the groups: 100.5 ± 42 for the T group and 92.2 ± 38 for the NT group, respectively. The average tourniquet usage was 69.4 ± 6 min, (*p* < 0.05) (Table 2).

At the first follow-up, we recorded significantly lower WOMAC pain scores for the NT group (7.3 ± 1.8 compared to 8.2 ± 3.1, *p* = 0.015). No other differences between the groups were seen at this follow-up. The VAS scale demonstrated a slight improvement for the NT group but was not significant from the statistical point of view. Both groups demonstrated improvements compared to preoperative results. Knee range of motion also showed significant improvements after the surgical procedure but with no significant differences between the groups. (Table 3) At the second follow-up, both groups showed significantly reduced knee pain and improved function. Postoperative results during the follow-ups are presented in Table 3. The average length of stay was 4.3 ± 2 days for the T group and 4.1 ± 3 days for the NT group, respectively (*p* = 0.58). Among those cases, 11 from the T group and 13 from the NT group were transferred to other institutions for rehabilitation.

Regarding complications, two patients from the T group and three from the NT group experienced arthrofibrosis, which required mobilization under anesthesia. In addition, two patients from the NT group and five from the T group experienced deep vein thrombosis that required therapeutic anticoagulation. Only one patient from the tourniquet group presented a wound complication, who required readmission after 15 days for a detailed assessment and who underwent antibiotic treatment for two weeks. No other complications or readmissions within 30 days were reported in our study.

## 4. Discussion

Tourniquet usage for TKAs has been under debate for a long time. However, the evidence regarding this topic from our country is lacking. Thus, we decided to develop this detailed study. The evidence from the literature is conflicting regarding blood loss, function and complications. Therefore, we opted for a prospective randomized trial for a more precise estimation of the postoperative outcome. 

The main finding of this paper was that both groups presented improvements regarding the WOMAC scores. We did not find statistically significant differences between the groups, except after 6 weeks, where we found better WOMAC pain scores for the NT group (*p* = 0.015). The same results have been reported by Guler et al., who recorded a similar level of knee function after 12 months [31]. On the other hand, Ejaz et al. reported faster recovery after 8 weeks but no difference after 12 months [33]. Regarding knee range of motion, we did not find any significant differences at any point of assessment. These results are also supported by other papers available [18,34]. However, Huang et al. found that patients treated without tourniquets had a greater range of motion at discharge [35].

Another parameter that we measured was the Visual Analogue Scale. Even though both groups demonstrated an improvement regarding pain, we did not find any significant differences between the groups. Despite our findings, Chen et al. reported that tourniquet usage for the entire TKA procedure was linked with significantly higher thigh pain [8]. However, our results are supported by Goel et al., who were not able to report any significant differences between the groups at any of the follow-ups [18].

We recorded a significantly higher amount of blood loss during the surgery (*p* < 0.05) and postoperatively (*p* < 0.05). Postoperative hemoglobin was also recorded as significantly lower in the tourniquet group (*p* < 0.05). Since the number of total knee replacements is rising, surgeons must take into account the amount of blood loss, as it may lead to an increased transfusion rate. This may increase the hospital costs for the procedure and also may increase the risk of complications [36,37,38]. Schnettler et al. discovered an increase in blood loss during tourniquet usage, stating that it could be due to hidden blood loss [39]. From our experience, we found that hemostasis is achieved more efficiently and methodically during the surgical approach without a tourniquet, with blood vessels being more visible compared to the other technique. On the other hand, Tai et al. found in a randomized trial that without a tourniquet, TKAs presented higher amounts of total blood loss [36]. Other papers suggest no significant differences regarding this subject [37,40,41]. In our opinion, those results could be highly dependent on the surgeon’s technique. We also think that, despite the significant differences between the groups, this may not be strongly significant from the clinical perspective if we take into account the functional results. In our institution, the tourniquet is deflated prior to incision closure in order to gain hemostasis, which is also the time when blood loss is predominant for this group. 

Another essential parameter for hospital costs is the operative time. We found significantly shorter operative room times for the NT group (*p* = 0.36), results which are also supported by other papers [37,42]. This difference may be caused by the time needed for the tourniquet application, and also by the hemostasis which needs to be achieved after deflation. The costs of 1 min of OR time reported in the literature are variable. Shippert et al. found an average cost of USD 66, including anesthesia [43]. Another paper reported costs between 36 and 37 USD [44]. According to Fletcher et al., the operating theatre time proved to cost around GBP 1200 per hour [45]. Nevertheless, all of these papers demonstrate that a significant reduction in operative time could be beneficial from a financial perspective.

The usage of tourniquets is also highly debated regarding postoperative complications. Tie et al. reported an increased rate of wound complications after prolonged tourniquet usage [46]. We also found reports in the literature about the increased risk of readmission, as well as an increased risk of thrombotic events [9,42,47,48]. Five cases from the T group and two from the NT group reported DVT. We also recorded two cases from the T group and three from the NT group with arthrofibrosis. Despite these reported complications, our paper was not powered to determine differences in complication rates correlated with tourniquet usage. Despite this fact, these results should be addressed.

Our study presents some limitations. Since the hospital stay is relatively short, we could not assess the hidden blood loss in both groups. We also admit that a one-year follow-up could have added additional data, despite the relevant information that we recorded. We also were not able to blind the surgeon to the allocation of the patient. Another limitation of this paper is that we used a high-pressure tourniquet, which is correlated with higher early surgical site pain levels compared to lower pressures [49,50]. However, in order to provide a better bloodless view of the surgical site, especially after cutting articular surfaces, we opted for a 320–350 mmHg pressure. We also deflated the tourniquet immediately after the implantation, which, according to Pinsornak et al., may help decrease local pain and the risk of complications [49]. We are considering the assessment of lower-pressure tourniquet usage upon the surgical outcome and potential complications, especially in the long term. Regarding complications, we consider it necessary for higher cohorts to be used in the future, in order to properly evaluate and correlate with the tourniquet usage properly.

## 5. Conclusions

Considering the result of our study, we found a significant decrease in bleeding after no tourniquet usage during total knee replacements, as well as a shorter operative time. On the other hand, based on the result of our study, the knee function demonstrated no significant differences between the groups. Additional studies are required to improve the quality of the evidence, in order to determine whether tourniquets should be used during TKAs, and to analyze the long-term effect of tourniquet use. Non-tourniquet usage provides good short-term results with less blood loss. We did not find a higher risk of complications, but further studies are necessary.

## Figures and Tables

**Table 1:** Baseline characteristics.

	Tourniquet	No Tourniquet	*p* Value
Age	68.4 ± 7.8	67.5 ± 8.1	0.43
Sex (m/f)	49/51	49/45	0.66
Preoperative hemoglobin	13.4 ± 1.4	13.3 ± 1.6	0.64
Flexion	74.8 ± 13.8	75.4 ± 15.3	0.77
Extension	−13.8 ± 14.3	−13.1 ± 15.1	0.74

**Table 2:** Intraoperative and postoperative parameters. *p* values with asterisks are statistically significant.

	Tourniquet	Non Tourniquet	*p* Value
Intraoperative bleeding (mL)	276 ± 109.2	245 ± 97.8	<0.05 *
Postoperative bleeding (mL)	353.44 ± 101.55	324.8 ± 151.65	<0.05 *
Postoperative Hemoglobin (g/dL)	11.23 ± 1.5	11.5 ± 1.3	<0.05 *
Operative Room time(min)	100.5 ± 42	92.2 ± 38	<0.05 *

* statistically significant.

**Table 3:** Postoperative results during follow-ups. *p* values with asterisks are statistically significant.

		Tourniquet	No Tourniquet	*p* Value
WOMAC Score Pain (0–20)	Preop	16.8 ± 5.09	18.4 ± 6.3	0.0525
	6 w	8.2 ± 3.1	7.3 ± 1.8	0.015 *
	6 m	7.8 ± 4.1	7.1 ± 2.5	0.15
WOMAC Score Stiffness (0–8)	Preop	6.3 ± 1.4	6.6 ± 1.9	0.21
	6 w	3.2 ± 1.1	2.9 ± 1.3	0.083
	6 m	2.8 ± 0.9	2.5 ± 1.1	0.038
WOMAC Score Function (0–68)	Preop	57.8 ± 10.1	56.9 ± 9.9	0.53
	6 w	23.6 ± 8.9	25.1 ± 8.8	0.26
	6 m	20.8 ± 8.1	21.3 ± 7.5	0.65
Flexion	6 w	103.4 ± 14.8	102.5 ± 16.8	0.69
	6 m	110.9 ± 9.8	111.2 ± 8.5	0.82
Extension	6 w	−8.1 ± 5.3	−9.3 ± 6.1	0.14
	6 m	−3.5 ± 2.4	−2.9 ± 3.1	0.13
VAS	6 w	3.79 ± 1.32	3.39 ± 1.50	0.052
	6 m	1.3 ± 1.01	1.41 ± 0.89	0.92

* statistically significant.

## Data Availability

All data generated or analyzed are included in this published article.

## Abbreviations

List of abbreviations.

TKA	Total Knee Arthroplasty
T group	Tourniquet group
NT group	No tourniquet group
VAS	Visual Analogue Scale
DVT	Deep Vain Thrombosis
WOMAC	Western Ontario and McMaster Universities Osteoarthritis Index
